# Prognostic impact of tumor microenvironment-related markers in patients with adenocarcinoma of the lung

**DOI:** 10.1007/s10147-022-02271-0

**Published:** 2022-11-14

**Authors:** Mayu Sugai, Naoki Yanagawa, Shunsuke Shikanai, Mitsumasa Osakabe, Makoto Maemondo, Hajime Saito, Tamotsu Sugai

**Affiliations:** 1 Department of Molecular Diagnostic Pathology, School of Medicine, Iwate Medical University, Yahaba-cho shiwa-gun, Iwate 028-3695 Japan; 2 Department of Respiratory Medicine, School of Medicine, Iwate Medical University, Yahaba-cho shiwa-gun, Iwate 028-3695 Japan; 3 Department of Thoracic Surgery, School of Medicine, Iwate Medical University, Yahaba-cho shiwa-gun, Iwate 028-3695 Japan

**Keywords:** Cancer-associated fibroblast, Immunohistochemistry, Lung adenocarcinoma, Podoplanin, ZEB1

## Abstract

Cancer-associated fibroblasts (CAFs) are a prominent component in the tumor microenvironment (TME), which plays an important role in lung carcinogenesis. Here, we investigated microenvironmental markers expressed by CAFs, including α-smooth muscle actin, CD10, podoplanin, fibroblast-specific protein 1, platelet-derived growth factor α and β, fibroblast-associated protein, tenascin-C, zinc finger E-box binding homeobox 1 (ZEB1), and twist-related protein 1 expression levels. We evaluated samples from 257 patients with lung adenocarcinoma (LAD) to assess the associations of CAF-related protein expression patterns with prognosis. LAD cases were stratified using cluster analysis. To determine the utility of prognostic markers in LAD, univariate and multivariate analyses were performed. LAD cases were classified into subgroups 1 and 2. Subgroup 2 was shown to be significantly correlated with disease-free and overall survival using univariate and multivariate analyses in this group. Upregulation of podoplanin was identified as a single prognostic marker in this study by univariate and multivariate analyses. In addition, ZEB1 overexpression was correlated with disease-free survival. Our current results suggested that the specific CAF phenotype (e.g., the expression pattern of CAF-related proteins) could predict outcomes in patients with LAD. In addition, podoplanin upregulation may predict outcomes in these patients.

**Supplementary Information:**

The online version contains supplementary material available at 10.1007/s10147-022-02271-0.

## Introduction

Lung cancer is one of the leading causes of cancer-related death worldwide [1, 2]. Lung cancer can be histologically classified into small cell lung cancer (SCLC) and non-SCLC (NSCLC), accounting for 15% and 85% of all lung cancers, respectively [2, 3]. Furthermore, NSCLC can be subclassified into adenocarcinoma, squamous cell carcinoma, and large-cell carcinoma [4, 5]. Lung adenocarcinoma (LAD) is the major subtype of NSCLC, accounting for approximately 50% of all NSCLC cases [5]. Despite rapid progress in clinical treatments for lung cancer, including surgery, chemotherapy, molecular targeted therapy, and immunotherapy, satisfactory outcomes for patients with LAD have not yet been achieved, and the 5-year survival rate is only 15% owing to drug resistance or poor responses to therapy [6].

The tumor microenvironment (TME) plays major roles in lung carcinogenesis and metastatic spread into the lymph nodes and distant organs [7–9]. The TME consists of two components, i.e., cancer cells and the surrounding cancer stroma [10]. According to this theory, tumor cells and CAFs within the TME synergistically enhance the metastatic potential of the tumor [7–10]. Although this phenomenon is still not fully understood, activation of CAF-related proteins is thought to occur in CAFs [10, 11]. We previously identified the important roles of CAFs in cancer progression in patients with LAD and in patients with adenocarcinomas derived from other organs (e.g., colorectal cancer and ovarian cancer) [10, 12]. CAFs strongly modulate therapy resistance, clinical outcomes, and disease progression, and the sensitivity of chemotherapy depends on the autonomous resistance of target cells owing to the negative impact of chemotherapy in the stimulation of CAFs, creating chemoresistance by releasing CAF-related proteins [13–17]. These results have important clinical implications because most chemosensitizing approaches have focused on evaluation of the molecular mechanisms involving CAFs [13–17].

In this study, we explored the expression patterns of CAF-related proteins present in LAD and assessed whether CAF-related proteins could affect outcomes in patients with LAD. Additionally, the impact of individual markers identified from our analysis of expression patterns in LAD was investigated. Overall, our study provided insights into the mechanisms through which CAFs promote tumor progression and established a potential strategy for overcoming therapy resistance in patients with LAD.

## Materials and methods

### Patients

In total, 257 cases of LAD were obtained from Iwate Medical University between 2010 and 2016. The World Health Organization (WHO) classification criteria [5] were used to establish histological classifications. Furthermore, International Association for the Study of Lung Cancer (IASLC) classifications [18] were employed to classify tumors as grade 1, 2, or 3. The percentage of each histological component was recorded in 5% increments according to the 2021 WHO classification. Briefly, all five major patterns recognized by the WHO, as well as non-traditional patterns, such as cribriform and fused glands (complex glandular patterns), were evaluated, and the proportions of each pattern within the tumor were calculated (totaling 100%) [18]. Additionally, tumor spread within air spaces (STAS) was identified according to the presence of micropapillary or solid clusters of single tumor cells that floated freely within air spaces beyond the edge of the tumor [19]. Tumor-infiltrating lymphocytes (TILs) were defined as previously reported [20]. Pathological stages (stage I in 149 patients, stages II and III in 108 patients) were determined according to the 8th Edition of the American Joint Committee on Cancer Staging Manual [21]. Detailed clinicopathological variables are shown in Table 1.

**Table 1:** Clinicopathological findings of lung adenocarcinoma cases and each subgroup

Characteristics			Subgroup 1		Subgroup 2		*p* value
Total	257		136		121		
Age median [range] (y)	69	[40–88]	69.5	[46–87]	69	[40–88]	0.8086
Sex							0.6161
Man	139	(54.1)	76	(55.9)	63	(52.1)	
Woman	118	(45.9)	60	(44.1)	58	(47.9)	
Smoking history							0.803
Yes	140	(54.5)	73	(53.7)	67	(55.4)	
No	117	(45.5)	63	(46.3)	54	(44.6)	
COPD							0.501
Yes	42	(16.3)	20	(14.7)	22	(18.2)	
No	215	(83.7)	116	(85.3)	99	(81.8)	
ILD							1
Yes	13	(5.1)	7	(5.1)	6	(5)	
No	244	(94.9)	129	(94.9)	115	(95)	
Adjuvant chemotherapy							< .0001
Yes	146	(56.8)	61	(44.9)	85	(70.2)	
No	111	(43.2)	75	(55.1)	36	(29.8)	
Histological subtype							< .0001
MIA	15	(5.8)	15	(11)	0	(0)	
Lepidic	6	(2.3)	6	(4.4)	0	(0)	
Papillary	138	(53.7)	61	(44.9)	77	(63.6)	
Acinar	51	(19.8)	31	(22.8)	20	(16.5)	
Solid	39	(15.2)	20	(14.7)	19	(15.7)	
Micropapillary	8	(3.1)	3	(2.2)	5	(4.1)	
IASLC grading system							< .0001
Grade1	21	(8.2)	21	(15.4)	0	(0)	
Grade2	160	(62.3)	78	(57.4)	82	(67.8)	
Grade3	76	(29.6)	37	(27.2)	39	(32.2)	
Pathologic Stage							< .0001
I	149	(58)	95	(69.9)	54	(44.6)	
II and III	108	(42)	41	(30.1)	67	(55.4)	
Invasive size, median [range] (mm)	22	[2–130]	20	[2–130]	24	[6–75]	0.0003
Lymph node metastasis							0.0001
Positive	88	(34.2)	32	(23.5)	56	(46.3)	
Negative	169	(65.8)	104	(76.5)	65	(53.7)	
Pleural invasion							0.0051
Positive	85	(33.1)	34	(25)	51	(42.1)	
Negative	172	(66.9)	102	(75)	70	(57.9)	
Lymphatic invasion							< .0001
Positive	57	(22.2)	17	(12.5)	40	(33.1)	
Negative	200	(77.8)	119	(87.5)	81	(66.9)	
Venous invasion							0.0397
Positive	61	(23.7)	25	(18.4)	36	(29.8)	
Negative	196	(76.3)	111	(81.6)	85	(70.2)	
STAS							0.0202
Positive	97	(37.7)	42	(30.9)	55	(45.5)	
Negative	160	(62.3)	94	(69.1)	66	(54.5)	
EGFR mutation							0.4541
Positive	117	(45.5)	65	(47.8)	52	(43)	
Negative	140	(54.5)	71	(52.2)	69	(57)	
TIL							0.0002
High	71	(27.6)	51	(37.5)	20	(16.5)	
Low	186	(72.4)	85	(62.5)	101	(83.5)	
Recurrence							< .0001
Yes	127	(49.4)	46	(33.8)	81	(66.9)	
No	130	(50.6)	90	(66.2)	40	(33.1)	
Outcome							< .0001
Death	77	(30)	25	(18.4)	52	(43)	
Survival	180	(70)	111	(81.6)	69	(57)	
Disease-free survival, median [range] (d)	1454	[40–4082]	1887	[40–4082]	823	[53–3619]	< .0001
Overall survival, median [range] (d)	1934	[119–4136]	2174.5	[127–4082]	1823	[119–4136]	0.0025

*COPD* chronic obstructive pulmonary disease, *ILD* interstitial lung disease, *MIA* minimally invasive adenocarcinoma, *IASLC* International Association for the Study of Lung Cancer, *STAS* spread through air spaces, *EGFR* epidermal growth factor receptor, *TILs* tumor-infiltrating lymphocytes

This study was approved by the Ethics Committee of Iwate Medical University School of Medicine (approval no. MH2021-047), and all patients provided written informed consent for participation. All study protocols and procedures were carried out based on the standards set by the Declaration of Helsinki.

### Assessment of overall survival (OS) and disease-free survival (DFS)

OS was evaluated based on lung cancer-specific survival, which was defined as cause of death from lung cancer. Additionally, DFS was assessed according to recurrence-free survival, excluding secondary cancers. DFS duration was evaluated according to the presence/absence of metastasis, measured 3–4 times/year during the follow-up period using computed tomography.

### Analysis of immunohistochemical data

The stromal fibroblastic compartment of each tumor was assessed to evaluate immunopositivity for α-SMA, fibroblast-associated protein (FAP), tenascin-C, podoplanin, CD10, platelet-derived growth factor receptor (PDGFR) α, PDGFRβ, fibroblast-specific protein 1 (FSP1), zinc finger E-box binding homeobox 1 (ZEB1), and twist-related protein 1 (TWIST1), while excluding inflammatory cells. For ZEB1 and TWIST1, cells were only considered positive when nuclear staining was observed, whereas for α-SMA, FAP, tenascin-C, podoplanin, CD10, PDGFRα, PDGFRβ, and FSP1, cells were considered positive when cytoplasmic staining was observed. Separate evaluations were performed to assess the immunostaining intensity and area. The immunostaining intensity for fusiform stromal cells was classified as negative, weak, moderate, or strong, and the immunostaining area for fusiform stromal cells was semiquantified (0%, 1–25%, 26–50%, or 51–100%). The combination of intensity and area was scored (Supplementary Table 2), and positivity was judged as a score of more than 4. All assessments were performed by expert diagnostic pathologists (N.Y., M.O., T.S.) blinded to the study endpoint. Discordant results were addressed in a discussion meeting, and a consensus was reached. The examined markers were previously identified as CAF- and epithelial–mesenchymal transition (EMT)-related markers [22].

### Hierarchical cluster analysis of CAF- and EMT-related markers

Samples were grouped based on immunohistochemistry results using hierarchical cluster analysis; maximal homogeneity for each group and the greatest difference between groups were determined using Cluster 3.0 software (bonsai.hgc.jp/ ~ mdehoon/software/cluster/software.htm), with clustering algorithm set to centroid linkage clustering.

Determination of sample size, post-surgery chemotherapy in patients with LAD, tissue microarray construction, and immunohistochemistry are described in the Supplementary Methods.

### Statistical analysis

Data analysis was performed using JMP Pro 16.1 software (SAS). Fisher’s exact tests were used for comparisons of the immunohistochemical positivity of each marker and clinicopathological findings with subgroups. Mann–Whitney *U* tests were performed for assessment of age distributions and invasive size among subgroups. Survival analysis was carried out using Kaplan–Meier analyses with log-rank tests. Findings were considered significant when the *p* value was less than 0.05. If multigroup comparisons were needed for statistical analysis, we used Bonferroni corrections.

Univariate and multivariate analyses were conducted with Cox proportional hazards models to identify significant differences for prediction of OS and DFS. The level of significance was set at *p* < 0.05, and the confidence interval (CI) was determined at the 95% level.

## Results

**Fig. 1:**
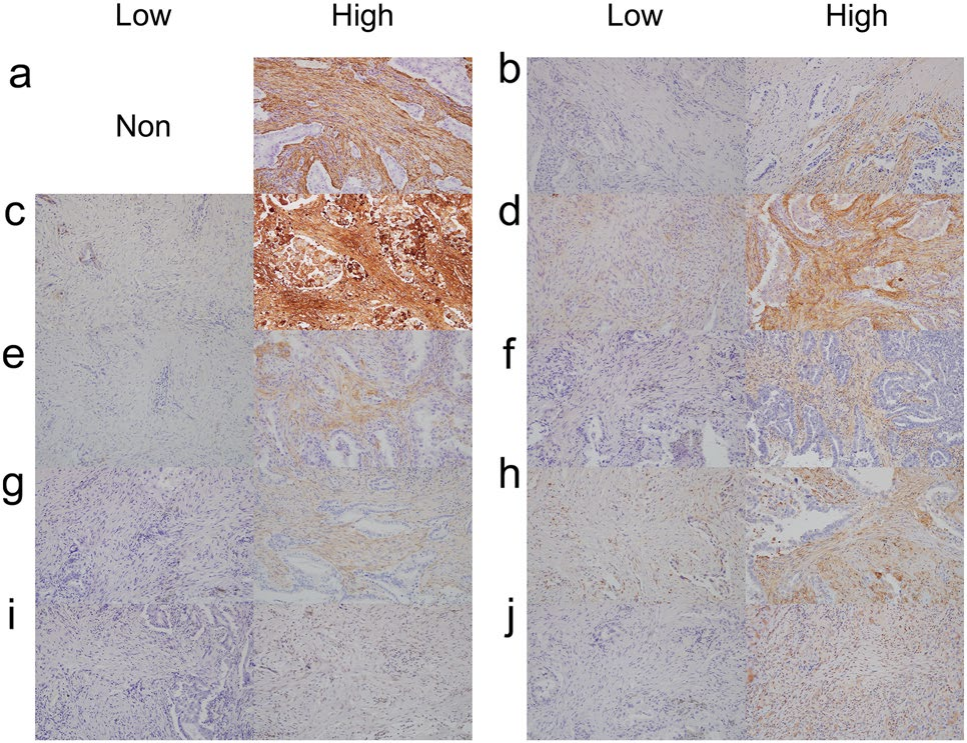
Representative features of immunohistochemical staining of the biological markers examined in this study based on expression score. **a** α-SMA. **b** FAP. **c** Tenascin-C. **d** Podoplanin. **e** CD10. (**f**) PDGFR-α. **g** PDGFR-β. **h** FSP1. **i** ZEB1. **j** TWIST1. Magnification: × 200

Representative histological features of LAD with strong desmoplasia are shown in Supplementary Fig. 1. Fig. 1 shows representative immunohistochemical features.

### Hierarchical clustering according to marker scores

**Fig. 2:**
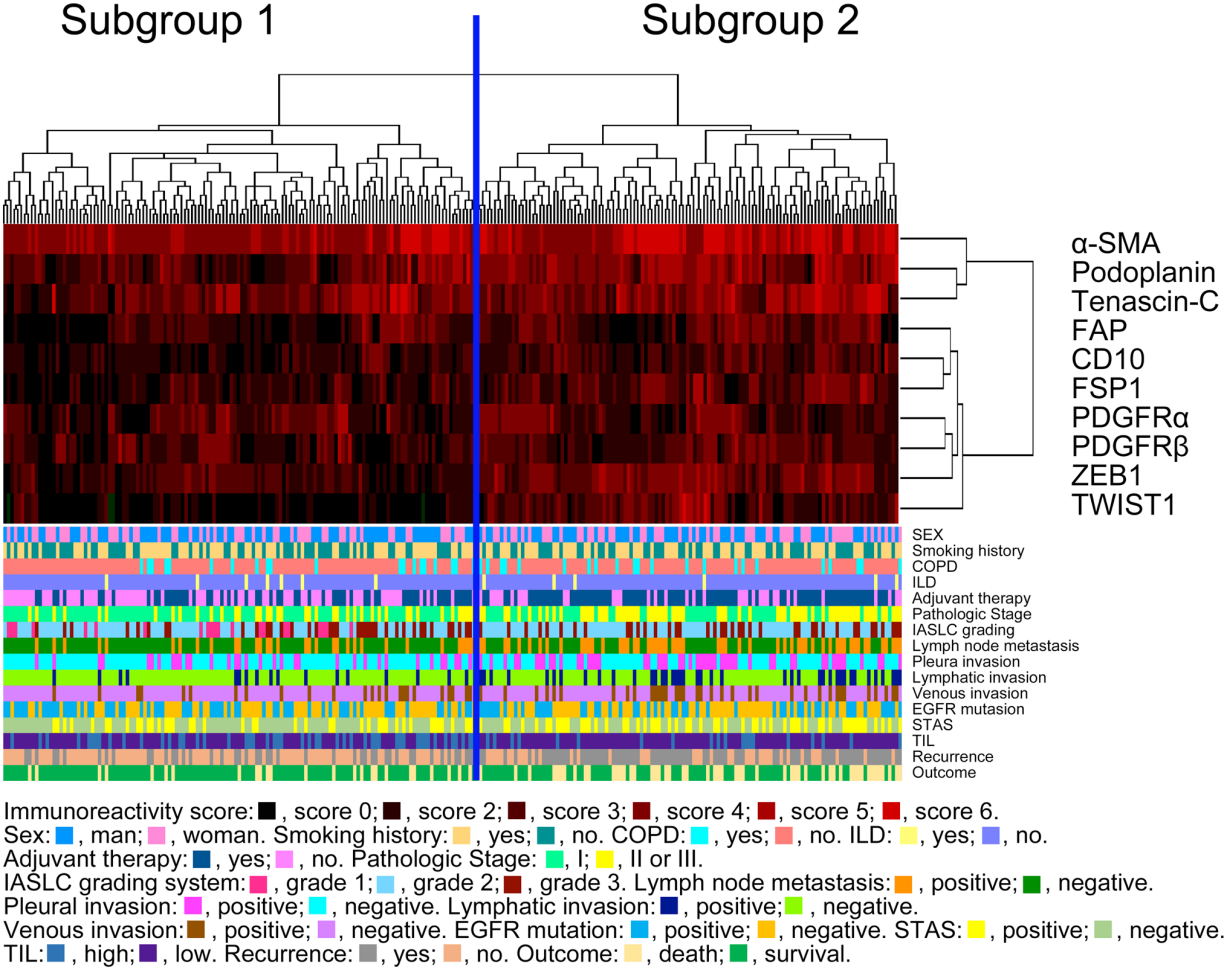
Hierarchical cluster analysis of patients with lung adenocarcinoma based on the expression patterns of cancer cells and cancer-associated fibroblast (CAF)-related proteins. The examined lung adenocarcinomas were subclassified into 2 subgroups.

Hierarchical clustering was carried out according to marker scores for assessment of differences in CAF and EMT marker expression patterns in patients with LAD. The analysis identified two distinct subgroups (Fig. 2; subgroups 1 and 2). Notably, chemotherapy treatment was more frequently reported in subgroup 2 than in subgroup 1. Furthermore, the frequencies of DFS and OS differed significantly between subgroups (subgroup 2 > subgroup 1; Table 1). Finally, significant differences between subgroups 1 and 2 were found for other clinicopathological factors, including pathological stage, IASLC grading, invasive size, lymph node metastasis, pleural invasion, lymphatic invasion, venous invasion, STAS, and TILs (Table 1). However, no differences in the frequencies of epidermal growth factor receptor (*EGFR*) mutations were found between subgroups 1 and 2.

Kaplan–Meier analyses showed that patients in subgroup 2 had reduced DFS compared with patients in subgroup 1 (*p* < 0.0001; Supplementary Fig. 2a). Moreover, patients in subgroup 2 showed decreased OS (*p* < 0.0001; Supplementary Fig. 2b).

### Association of clinicopathological findings and subgroups with patient survival

Six factors (i.e., smoking history, pathological stage, IASLC grading system, lymphatic invasion, venous invasion, and subgroup) were found to be associated with DFS (Table 2-a), and 3 factors (smoking history, pathological stage, and subgroup) were retained (Table 2-b). Eight factors (i.e., sex, smoking history, pathological stage, IASLC grading system, lymphatic invasion, venous invasion, *EGFR* mutation, and subgroup) were found in univariate analysis of OS (Table 2-c), but only pathological stage, *EGFR* mutation, and subgroup were retained after multivariate analysis (Table 2-d). Overall, *EGFR* mutation status was correlated with OS in both univariate and multivariate analyses, but was not correlated with DFS in univariate analysis (Table 2).

**Table 2:** Association of clinicopathological variables and subgroups with disease-free survival (a and b) and overall survival (c and d) in univariate and multivariate analyses

		A. Univariate analysis			B. Multivariate analysis			C. Univariate analysis			D. Multivariate analysis		
		HR	95% CI	*p* value	HR	95% CI	*p* value	HR	95% CI	*p* value	HR	95% CI	*p* value
Sex	Man versus woman	1.248	0.879–1.771	0.2146				1.810	1.140–2.875	0.0119	1.291	0.697–2.391	0.4175
Age		1.000	0.981–1.020	0.9808				1.018	0.993–1.045	0.1657			
Smoking history	Yes versus no	1.442	1.011–2.056	0.0432	1.550	1.076–2.234	0.0187	1.942	1.214–3.108	0.0057	1.477	0.800–2.728	0.2123
Pathologic stage	II or III versus I	4.741	3.260–6.894	< 0.0001	3.253	2.151–4.919	< 0.0001	4.980	3.007–8.247	< 0.0001	3.373	1.925–5.911	< 0.0001
IASLC grading system	2 versus 1	6.456	2.784–26.29	0.0092	2.696	0.642–11.33	0.1757	5.983	0.822–43.56	0.0773	2.619	0.347–19.74	0.3502
	3 versus 1	11.47	1.237–47.22	0.0007	3.142	0.727–13.59	0.1254	14.67	2.012–106.9	0.0081	3.868	0.503–29.76	0.1938
Lymphatic invasion	Positive versus negative	2.883	1.991–4.173	< 0.0001	1.313	0.861–2.002	0.2055	2.958	1.871–4.675	< 0.0001	1.508	0.878–2.591	0.1368
Venous invasion	Positive versus negative	2.355	1.626–3.409	< 0.0001	1.292	0.854–1.954	0.2253	1.919	1.195–3.082	0.007	0.994	0.590–1.673	0.9809
EGFR mutation	Positive versus negative	1.008	0.712–1.429	0.9625				0.537	0.336–0.859	0.0094	0.503	0.306–0.827	0.0068
Subgroup	2 versus 1	2.545	1.768–3.663	< 0.0001	1.732	1.182–2.537	0.0048	2.747	1.703–4.431	< 0.0001	1.849	1.116–3.061	0.017

*IASLC* International Association for the Study of Lung Cancer, *EGFR* epidermal growth factor receptor, *HR* hazard ratio, 95% *CI* 95% confidence interval

### Comparison of individual markers for each subgroup

**Fig. 3:**
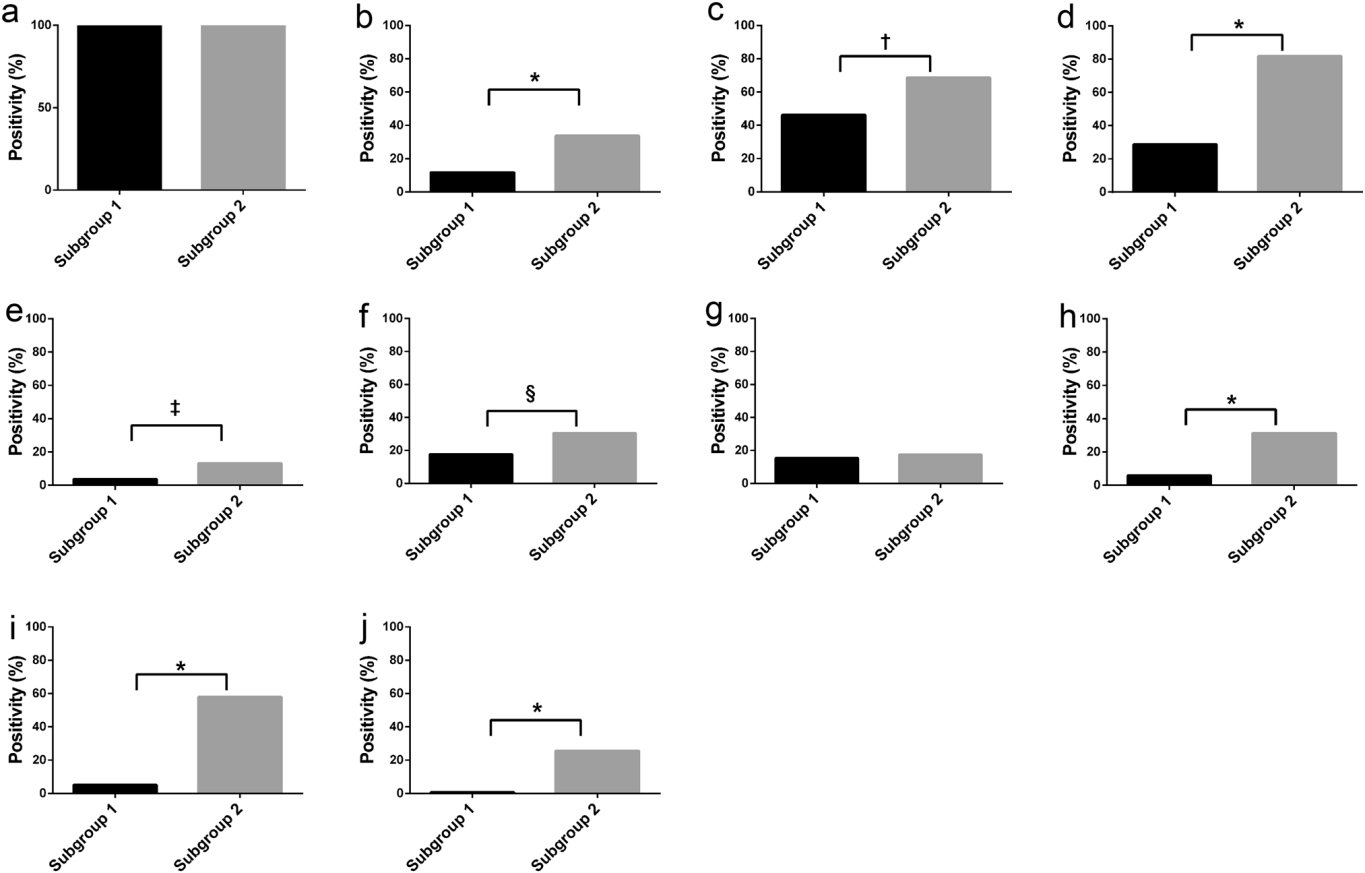
Expression level of each marker in lung adenocarcinoma. **a** α-SMA. **b** FAP. **c** Tenascin-C. **d** Podoplanin. **e** CD10. **f** PDGFR-α. **g** PDGFR-β. **h** FSP1. **i** ZEB1. **j** TWIST1. *, *p* =  < 0.0001; †, *p* = 0.0004; ‡, *p* = 0.0006; §, *p* = 0.0186

In CAFs, FAP (*p* < 0.0001), tenascin-C (*p* = 0.0004), podoplanin (*p* < 0.0001), CD10 (*p* = 0.0006), PDGFRα (*p* = 0.0186), FSP1 (*p* < 0.0001), TWIST1 (*p* < 0.0001), and ZEB1 (*p* < 0.0001) positivity ratios were significantly higher in subgroup 2 than in subgroup 1 (Fig. 3).

### Association of clinicopathological findings and various markers with patient survival

Next, we assessed whether clinicopathological variables and marker expression patterns could independently predict clinical outcomes in patients with LAD using multivariate analysis followed by Cox proportional hazard analysis of mortality risk using significant univariate correlators (predictors). αSMA expression was excluded because this marker was expressed in all examined cases. We examined the associations of pathological findings and each marker with DFS. Although pathological stage, IASLC grading system, and FAP, tenascin-C, podoplanin, ZEB1, and TWIST1 expression were correlated with DFS in univariate analysis (Table 3-a), only pathological stage, podoplanin, and ZEB1 expression were retained in multivariate analysis, even after adjusting for other variables (Table 3-b). Furthermore, univariate analysis (Table 3-c) identified 8 factors, including pathological stage, IASLC grading system, *EGFR mutation*, FAP, tenascin-C, podoplanin, PDGFRα, and ZEB1 expression, as being associated with OS. However, pathological stage, *EGFR* mutation, and podoplanin expression were retained in multivariate analysis (Table 3-d).

**Table 3:** Association of clinicopathological variables and individual markers with disease-free survival (a and b) and overall survival (c and d) in univariate and multivariate analyses

		A. Univariate analysis			B. Multivariate analysis			C. Univariate analysis			D. Multivariate analysis		
		HR	95% CI	*p* value	HR	95% CI	*p* value	HR	95% CI	*p* value	HR	95% CI	*p* value
Pathologic stage	II or III versus I	4.741	3.26–6.894	< 0.0001	3.587	2.375–5.418	< 0.0001	4.980	3.007–8.247	< 0.0001	3.625	2.05–6.411	< 0.0001
IASLC grading system	2 versus 1	6.455	2.784–26.29	0.0092	2.378	0.560–10.10	0.2404	5.983	0.822–43.56	0.0773	2.151	0.279–16.60	0.4625
	3 versus 1	11.47	1.237–47.22	0.0007	3.010	0.691–13.12	0.1423	14.67	2.012–106.9	0.0081	3.682	0.472–28.71	0.2135
EGFR mutation	Positive versus negative	1.008	0.712–1.429	0.9625				0.537	0.336–0.859	0.0094	0.592	0.364–0.963	0.0348
αSMA*	Positive versus negative												
FAP	Positive versus negative	1.612	1.092–2.381	0.0163	1.409	0.924–2.148	0.1111	2.041	1.272–3.275	0.0031	1.532	0.917–2.56	0.1033
Tenascin-C	Positive versus negative	1.928	1.333–2.787	0.0005	1.256	0.852–1.851	0.2506	2.062	1.272–3.342	0.0033	1.131	0.68–1.882	0.6344
Podoplanin	Positive versus negative	2.650	1.809–3.880	< 0.0001	1.857	1.223–2.820	0.0037	3.203	1.907–5.381	< 0.0001	1.780	1.016–3.12	0.0438
CD10	Positive versus negative	1.068	0.575–1.983	0.8346				1.273	0.612–2.648	0.5176			
PDGFRα	Positive versus negative	0.706	0.458–1.086	0.1133				0.487	0.257–0.922	0.0273	0.635	0.33–1.222	0.1737
PDGFRβ	Positive versus negative	0.707	0.424–1.178	0.1833				0.628	0.314–1.260	0.1904			
FSP1	Positive versus negative	1.398	0.907–2.154	0.1288				1.434	0.826–2.487	0.2000			
ZEB1	Positive versus negative	2.366	1.662–3.370	< 0.0001	1.596	1.075–2.370	0.0205	2.231	1.423–3.497	0.0005	1.386	0.848–2.264	0.1927
TWIST1	Positive versus negative	1.649	1.040–2.615	0.0334	0.736	0.437–1.238	0.2483	1.505	0.829–2.733	0.1791			

*IASLC* International Association for the Study of Lung Cancer, *EGFR* epidermal growth factor receptor, *α-SMA* α-smooth muscle actin, *FAP* fibroblast-activating protein, *PDGFR* platelet-derived growth factor receptor, *FSP1* fibroblast-specific protein 1, *ZEB1* zinc finger E-box binding homeobox 1, *TWIST1* TWIST homolog 1 gene, *HR* hazard ratio, 95% *CI* 95% confidence interval. *Expression of αSMA was excluded in the current study, given that this marker was expressed in all of the examined cases

### Relationships among EMT- and CAF-related markers

We examined the associations of positive expression of EMT-related markers (ZEB1 and TWIST) with positive expression of CAF-related markers. However, positive expression of ZEB1 and TWIST was not associated with positive expression of CAF-related markers (Supplementary Table 3).

### Association of positive expression of ZEB1 with histological type

No significant differences in the positive expression of ZEB1 were observed among the six histological types, i.e., MIA, lepidic, papillary, acinar, solid, and micropapillary types (Supplementary Fig. 3).

### Relationship between podoplanin expression and TILs in tumor tissues

We examined the association of TIL grade (low and high) with podoplanin expression. However, no correlations were observed (Supplementary Table 4).

### Association of patient prognosis with stages I and II/III LAD

We investigated the association of LAD subgroups with prognosis in patients with stages I and II/III LAD. Subgroup 2 (stage I LAD) had a poor prognosis compared with subgroup 1 (stage I LAD). Subgroups 1 and 2 were not correlated with prognosis in patients with stage II/III LAD (Supplementary Fig. 2c–f).

### High frequency of podoplanin expression in LAD according to stage

The frequency of high podoplanin expression was significantly increased in stage III LAD compared with stage I LAD. No association was observed for stage II LAD (Supplementary Fig. 4).

### Association of podoplanin expression with stages I and II/III LAD

High podoplanin expression was correlated with OS and DFS in patients with stage I LAD. Additionally, although high podoplanin expression was correlated with DFS in patients with stage II/III LAD, no association with OS was observed for stage II/III LAD (Supplementary Fig. 5).

## Discussion

CAFs are a heterogeneous population of cells, and this heterogeneity may depend on the numerous cellular precursors of CAFs [23–25]. Furthermore, the heterogeneity of activated fibroblasts could lead to the phenotypic heterogeneity of CAFs, manifesting as diverse biological marker expression on specific CAFs [24–26]. Several markers with differential expression in CAFs can be used to examine CAF functions [10, 11, 27]. However, none of these markers are commonly expressed by all CAFs, highlighting the heterogeneity of CAFs described herein as the expression pattern of CAF-related proteins. Thus, the expression pattern of CAF-related markers (the CAF phenotype) may affect cancer progression, metastasis, and prognosis in patients with LAD [11, 24]. In this study, we found that subgroup 2, which was stratified according to cluster analysis, may correspond to a poor prognostic CAF phenotype. However, defining a functional population of CAFs using multiple markers remains challenging owing to the diversity of CAF markers. Future studies may require in vivo models to interpret the heterogeneity of CAFs in the context of CAF-related marker expression, pathological stage, and patient prognosis.

Human podoplanin is a 38-kDa type-1 transmembrane glycoprotein consisting of 162 amino acids, 9 of which form the intracellular domain [28]. Although podoplanin (also known as D2-40) has been often used as an endothelial marker in surgical pathology, it is also expressed in various cell types, including mesothelial cells, follicular dendritic cells, and CAFs. In this study, we found that podoplanin upregulation was correlated with outcomes in patients with LAD. In addition, the current result showed that podoplanin upregulation may be helpful to predict patient prognosis with stage I LAD. Consistent with this, recruitment of podoplanin-positive CAFs is correlated with poor outcomes in patients with LAD [28–32]. Furthermore, podoplanin expression in CAFs promotes LAD engraftment into SCID mice (which have a genetic immune deficiency owing to an autosomal recessive mutation), thereby affecting B and T cells [28, 32]. Accordingly, podoplanin-expressing CAFs create a supportive microenvironment that promotes tumor progression [28]. Notably, CAFs expressing podoplanin enhance the local invasion of cancer cells owing to invasion into the collagen matrix [28], and inhibition of ROCK signaling by podoplanin knockdown in CAFs decreases the invasion ability of CAFs [28]. This finding suggested that local invasion of cancer cells may depend on the invasion ability of a certain subtype of recruited CAFs in the tumor tissue [28]. Therefore, treatment with a ROCK inhibitor may significantly decrease the invasion area and the number of invaded cancer cells owing to podoplanin overexpression-dependent enhancement of RhoA activity in CAFs [28]. Taken together, findings from the current study and previous studies [28–32] suggest that podoplanin-positive CAFs may promote tumor progression, thereby decreasing survival.

The EMT-transcription factor zinc finger/homeodomain proteins ZEB1 and ZEB2 can act as transcriptional activators by binding to histone acetyl-transferases p300/pCAF [33]. ZEB1 and ZEB2 overexpression has been found in several human cancers, including NSCLC [33]. Moreover, increased ZEB1 expression is associated with tumor grade in LAD [34, 35] and ZEB1 promotes colorectal and breast cancer metastasis [36]. In lung cancer cell lines, ZEB1 is inversely correlated with E-cadherin expression and facilitates anchorage-independent colony formation [33, 36]. In this study, ZEB1 upregulation was found to be correlated with DFS in LAD. Thus, ZEB1 may have an important role in the pathogenesis of LAD and ZEB1 expression and EMT induction may be closely associated with the tumorigenesis of LAD.

We examined the relationship of positive expression of EMT-related markers (ZEB1 and TWIST) with positive expression of each CAF-related marker. Such associations may be interesting when evaluating the roles of CAFs in tumor progression and metastasis. Our findings suggested that the EMT phenomenon was not enforced by the examined CAF-related proteins, and vice versa. However, we showed that both EMT- and CAF-related proteins played crucial roles in prediction of prognosis in patients with LAD. Moreover, although we compared the positive expression of ZEB1, a major EMT-related protein, with each histological type, no associations were found. Accordingly, positive expression of ZEB1, which is a predictive marker for DFS, was not associated with any histological type.

TILs reflect adaptive antitumor immune responses in cancer and are generally associated with favorable prognosis in lung cancer. Accordingly, we evaluated the association of high-grade TILs with positive podoplanin expression, which was found to be an excellent prognostic marker in LAD in the current study. However, we found no associations. This finding suggested that high podoplanin expression was not associated with suppression of immunoresponses occurring in tumor tissues.

To evaluate the cause and effect relationships between this subgroup classification and prognosis, we investigated the association of each subgroup with prognosis in patients with stages I and II/III LAD. However, although subgroup 2 (stage I) was associated with a poor prognosis, we could not identify the association of subgroup 2 with OS and DFS in patients with stage II/III LAD. Thus, subgroup 2 may be related to prognosis only in patients with stage I disease. Importantly, stage II/III LAD is affected by various prognostic factors, including lymphatic and venous invasion, pleural invasion, and absence of lepidic growth, which are frequently found in advanced-stage disease [37–39].

There were some limitations to this study. First, although the first cohort was large, validation studies in a second cohort were not carried out. However, because the current cohort was large, we expect that our data concerning outcomes in patients with LAD were reliable. Second, heterogeneous expression of CAF- and EMT-related markers complicates the immunohistochemical analysis. In this study, immunohistochemical expression was evaluated in strong invasive regions of tumor samples. These invasive areas are considered appropriate for obtaining reproducible results to assess the roles of CAFs in LAD. Third, it may be difficult to apply the findings of our subgroup analysis to actual cases. However, we suggest that these subgroup findings may play critical roles in prediction of outcomes in patients with LAD. Finally, we used selected markers to evaluate the expression of CAF-related proteins. Although subjective results may be expected, the markers used in this study were considered reliable and reproducible for identification of the biological characteristics of CAFs. Therefore, we believe that subjective results were avoided and that we consequently obtained novel findings to evaluate lung carcinogenesis.

In conclusion, we examined the CAF phenotype, which is described herein as an expression pattern of CAF-related proteins, to identify whether this CAF phenotype was associated with prognosis in patients with LAD. As a result, we found that a specific CAF phenotype (here, subgroup 2) was correlated with prognosis. Second, we found that individual CAF-related markers were closely associated with clinical outcomes in these patients. Accordingly, these results implied that podoplanin upregulation may predict prognosis in patients with LAD as well. Finally, podoplanin may be a critical target gene for the treatment of LAD. However, further studies are needed to confirm these results.

## Supplementary Information

Below is the link to the electronic supplementary material.

Supplementary file1 Representative histological features of the strong desmoplastic reaction in lung adenocarcinoma (H.E. staining). Magnification: 200× (TIF 11450 KB)

Supplementary file2 Kaplan-Meier analyses of patient survival. a. Disease-free survival in each subgroup. b. Overall survival in each subgroup. c. Disease-free survival for stage I LAD in each subgroup. d. Overall survival for stage I LAD in each subgroup. e. Disease-free survival for stage II/III LAD in each subgroup. f. Overall survival for stage II/III LAD in each subgroup (TIF 767 KB)

Supplementary file3 Association of positive expression of ZEB1 with each histological type. p = 0.3223 (TIF 962 KB)

Supplementary file4 High frequency of podoplanin expression in stages I, II, and III LAD. *, p = 0.0054 (TIF 1455 KB)

Supplementary file5 Association of podoplanin expression with LAD in the entire cohort and in patients with stages I and II/III disease. a. Disease-free survival in the entire cohort. b. Overall survival in the entire cohort. c. Disease-free survival in patients with stage I disease. d. Overall survival in patients with stage I disease. e. Disease-free survival in patients with stage II/III disease. f. Overall survival in patients with stage II/III disease (TIF 764 KB)

Supplementary file6 (DOCX 22 KB)

Supplementary file7 (DOCX 21 KB)

## Data Availability

The datasets used and/or analyzed during the current study are available from the corresponding author on reasonable request.

## Abbreviations

CAF: Cancer-associated fibroblast
TMA: Tissue microarray
FSP1: Fibroblast-specific protein 1
PFDFR: Platelet-derived growth factor receptor
FAP: Fibroblast-associated protein
LAD: Lung adenocarcinoma
NSCLC: Non-small cell lung cancer
SCLC: Small cell lung cancer
ZEB1: Zinc finger E-box binding homeobox 1
TWIST1: Twist-related protein 1
