# Supplementary figures and images for: Prognostic impact of tumor microenvironment-related markers in patients with adenocarcinoma of the lung
Source: Int J Clin Oncol. 2022 Nov 14;28(2):229–39. doi: 10.1007/s10147-022-02271-0 (PMC9889427; doi:10.1007/s10147-022-02271-0)

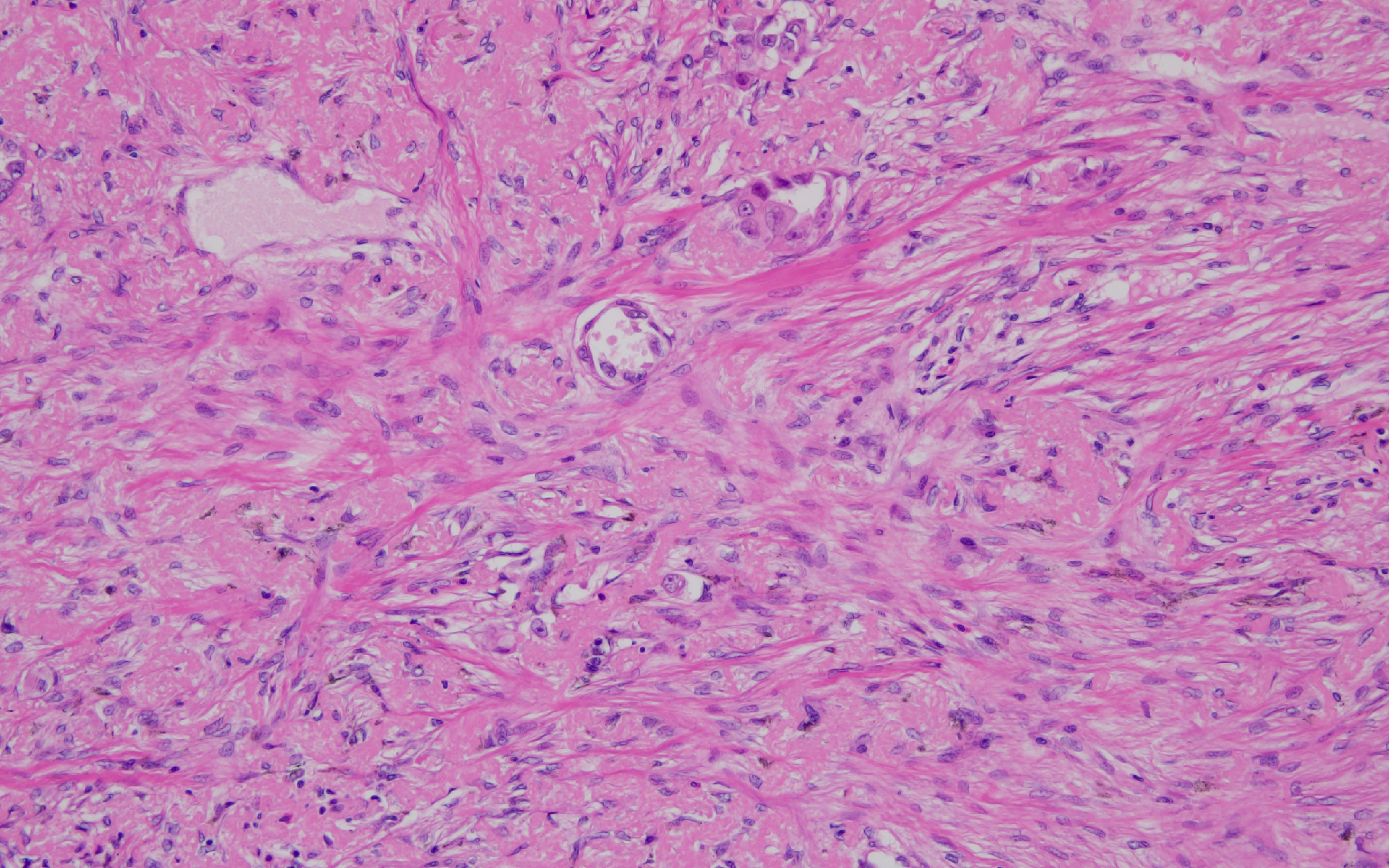

Supplement: Supplementary file 1 — Supplementary file1 Representative histological features of the strong desmoplastic reaction in lung adenocarcinoma (H.E. staining). Magnification: 200× (TIF 11450 KB) [file 10147_2022_2271_MOESM1_ESM.tif]

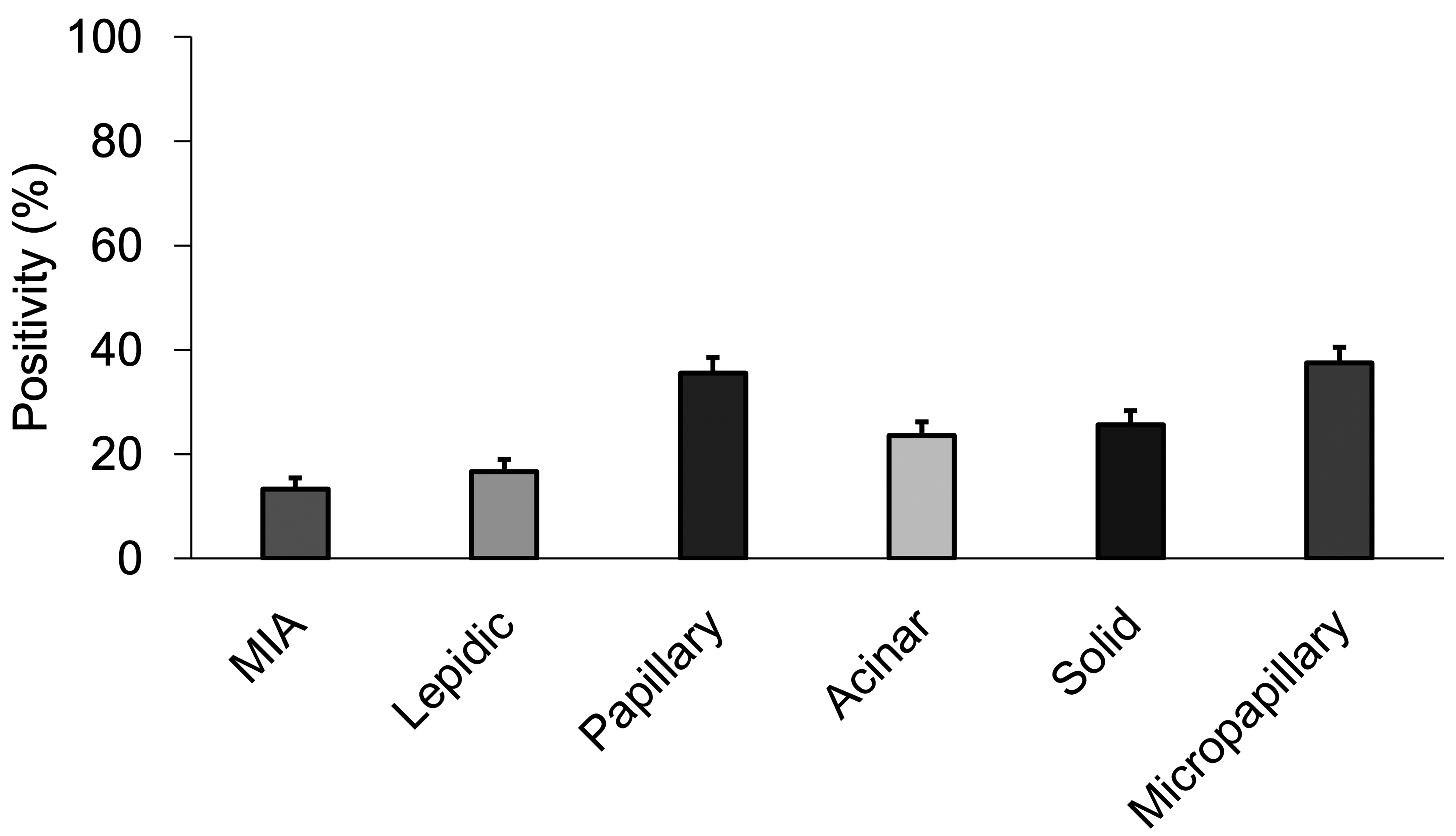

Supplement: Supplementary file 3 — Supplementary file3 Association of positive expression of ZEB1 with each histological type. p = 0.3223 (TIF 962 KB) [file 10147_2022_2271_MOESM3_ESM.tif]

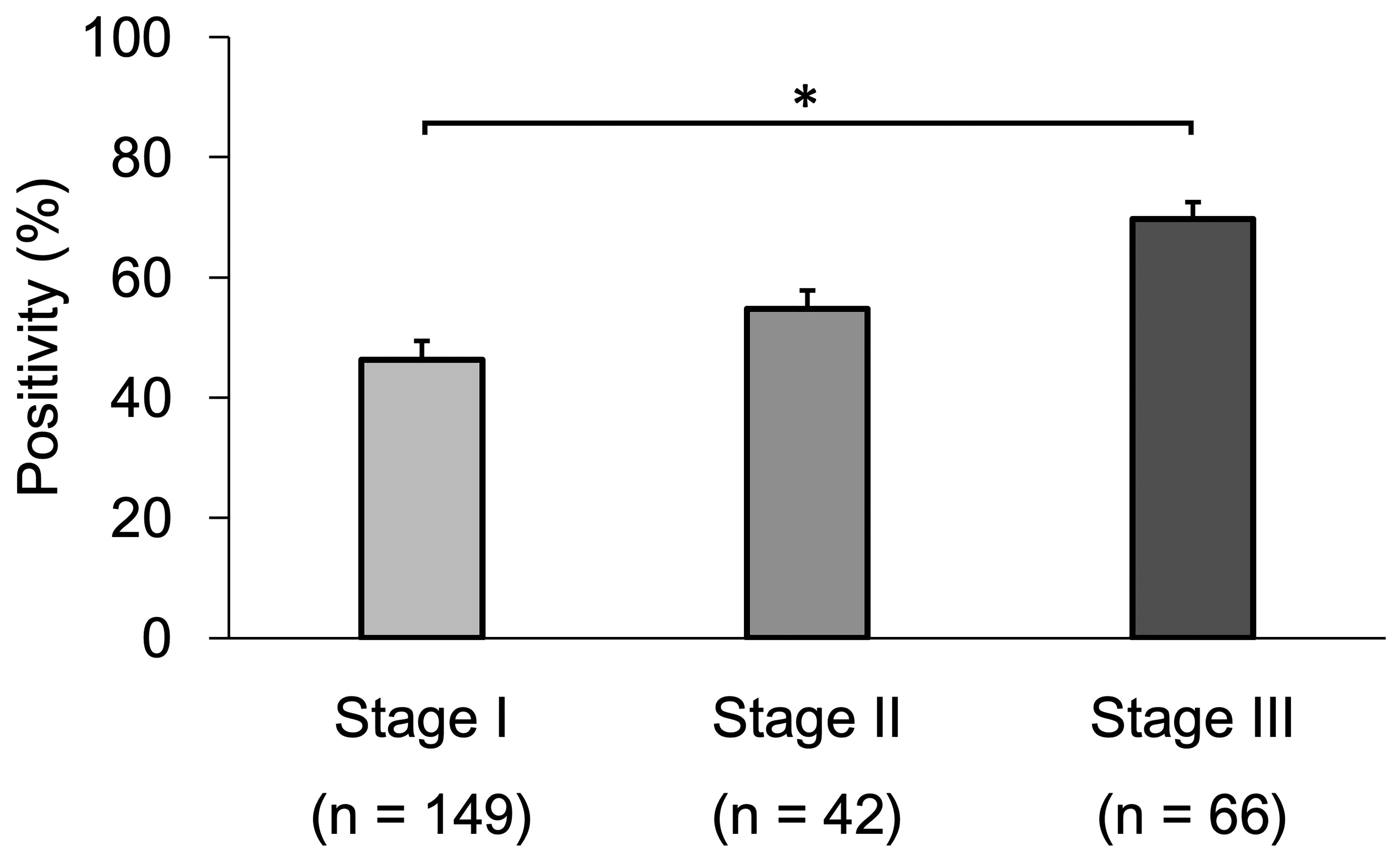

Supplement: Supplementary file 4 — Supplementary file4 High frequency of podoplanin expression in stages I, II, and III LAD. *, p = 0.0054 (TIF 1455 KB) [file 10147_2022_2271_MOESM4_ESM.tif]
