# Supplementary material for: Prognostic impact of tumor microenvironment-related markers in patients with adenocarcinoma of the lung
Source: Int J Clin Oncol. 2022 Nov 14;28(2):229–39. doi: 10.1007/s10147-022-02271-0 (PMC9889427; doi:10.1007/s10147-022-02271-0)
Supplement: Supplementary file 6 — Supplementary file6 (DOCX 22 KB) [file 10147_2022_2271_MOESM6_ESM.docx]

Supplementary Table 1. Immunohistochemical markers for CAFs and the EMT.

| Antibody target | Clone | Source | Dilution | Treatment |
| --- | --- | --- | --- | --- |
| α-SMA | 1A4 | Dako | Ready to use | Heat retrieval (pH 9.0) |
| FAP | EPR20021 | Abcam | 1:250 | Heat retrieval (pH 9.0) |
| Tenascin-C | 4F10TT | IBL | 1:200 | Heat retrieval (pH 9.0) |
| Podoplanin | D2-40 | Dako | Ready to use | Heat retrieval (pH 9.0) |
| CD10 | 56C6 | Dako | Ready to use | Heat retrieval (pH 9.0) |
| PDGFRα | poly | Cell Signaling Technology | 1:100 | Heat retrieval (pH 9.0) |
| PDGFRβ | 28E | Cell Signaling Technology | 1:50 | Heat retrieval (pH 9.0) |
| FSP1 | Poly | Dako | 1:400 | Heat retrieval (pH 6.0) |
| ZEB1 | Poly | Sigma-Aldrich | 1:200 | Heat retrieval (pH 6.0) |
| TWIST1 | Twist2C1a | Abcam | 1:200 | Heat retrieval (pH 9.0) |

α-SMA, α-smooth muscle actin; FAP, fibroblast-associated protein; PDGFR, platelet-derived growth factor receptor; FSP1, fibroblast-specific protein 1; ZEB1, zinc finger E-box binding homeobox 1; TWIST1, twist homolog 1 gene.

Supplementary Table 2. Scoring method.

|  |  | Staining area (%) | | | |
| --- | --- | --- | --- | --- | --- |
|  |  | 0 (0%) | 1 (1–25%) | 2 (26–50%) | 3 (51–100%) |
|  | 1. Negative | Score 0 | Score 0 | Score 0 | Score 0 |
| Staining  intensity | 1. Weak | Score 0 | Score 2 | Score 3 | Score 4 |
|  | 1. Moderate | Score 0 | Score 3 | Score 4 | Score 5 |
|  | 1. Strong | Score 0 | Score 4 | Score 5 | Score 6 |

Supplementary Table 3. Association of positive expression of EMT-related markers (ZEB1 and TWIST) with positive expression of CAF-related markers

| EMT marker | Number of positive cases (%) | | CAF marker | Number of positive cases (%) | | Κ [95% CI] | |
| --- | --- | --- | --- | --- | --- | --- | --- |
| ZEB1 | 77 | (30) | αSMA | 100 | (100) | - | - |
|  |  |  | FAP | 57 | (22.2) | 0.139 | [0.012, 0.265] |
|  |  |  | Tenascin C | 146 | (56.8) | 0.166 | [0.064, 0.268] |
|  |  |  | Podoplanin | 138 | (53.7) | 0.206 | [0.101, 0.311] |
|  |  |  | CD10 | 21 | (8.2) | 0.017 | [-0.078, 0.111] |
|  |  |  | PDGFRα | 61 | (23.7) | 0.054 | [-0.07, 0.177] |
|  |  |  | PDGFRβ | 42 | (16.3) | 0.073 | [-0.047, 0.192] |
|  |  |  | FSP1 | 46 | (17.9) | 0.256 | [0.13, 0.382] |
| TWIST | 32 | (12.5) | αSMA | 100 | (100) | - | - |
|  |  |  | FAP | 57 | (22.2) | 0.078 | [-0.049, 0.205] |
|  |  |  | Tenascin C | 146 | (56.8) | 0.068 | [-0.002, 0.138] |
|  |  |  | Podoplanin | 138 | (53.7) | 0.189 | [0.117, 0.261] |
|  |  |  | CD10 | 21 | (8.2) | 0.058 | [-0.082, 0.198] |
|  |  |  | PDGFRα | 61 | (23.7) | 0.088 | [-0.038, 0.213] |
|  |  |  | PDGFRβ | 42 | (16.3) | 0.056 | [-0.077, 0.189] |
|  |  |  | FSP1 | 46 | (17.9) | 0.128 | [-0.013, 0.269] |

EMT, epithelial-mesenchymal transition; CAF, cancer-associated fibroblast; Κ, kappa coefficient; ZEB1, Zinc finger E-box binding homeobox 1; TWIST1, TWIST homolog 1 gene; α-SMA, α-smooth muscle actin; FAP, fibroblast-activating protein; PDGFR, platelet-derived growth factor receptor; FSP1, fibroblast-specific protein 1.

Supplementary Table 4. Relationship between podoplanin expression and TILs in tumor tissues

|  | TILs | |  |
| --- | --- | --- | --- |
|  | Low | High | *p* value |
| Podoplanin |  |  |  |
| Negative | 86 | 33 | N.S. |
| Positive | 100 | 38 |  |

TILs, tumor-infiltrating lymphocytes; N.S., not significant.
