# Supplementary material for: Prognostic impact of tumor microenvironment-related markers in patients with adenocarcinoma of the lung
Source: Int J Clin Oncol. 2022 Nov 14;28(2):229–39. doi: 10.1007/s10147-022-02271-0 (PMC9889427; doi:10.1007/s10147-022-02271-0)
Supplement: Supplementary file 7 — Supplementary file7 (DOCX 21 KB) [file 10147_2022_2271_MOESM7_ESM.docx]

***Determination of sample size***

Using the Freedman method (R 4.2.0 software), we calculated the sample size needed to assess patient outcomes as at least 240 cases. Consistent with other clinical studies, we set the statistical power (detection power) to 0.8.

***Postsurgery chemotherapy in patients with LAD***

Postsurgery, tegafur uracil was administered to 66 of 146 patients with LAD, whereas 63 of 146 patients received platinum-doublet chemotherapy, including carboplatin plus gemcitabine, tegafur/gimeracil/oteracil, or paclitaxel. Twelve patients with LAD received radiotherapy, and 5 patients received epidermal growth factor receptor (*EGFR*) tyrosine kinase inhibitors (TKIs). The remaining 111 patients did not receive any postsurgery chemotherapy.

Whether chemotherapy was administered was determined according to the Guidelines for the Diagnosis and Treatment of Lung Cancer [1]. Regimens, including CBDCA plus gemicitabine, tegafur/gimeracil/oteracil, or paclitaxel, were administered to preserve renal function according to the treatment plan established by the Department of Thoracic Surgery of our institution. Administration of *EGFR* TKIs was performed according to the decision of physicians from the Department of Thoracic Surgery of our institution.

***Tissue microarray (TMA) construction***

Paraffin-embedded tissues were stored at room temperature before use in TMA construction, which was carried out using a manual tissue array (Azumaya Co., Tokyo, Japan). After creating TMAs, serial three-micron-thick tissue sections were cut and placed on charged slides. Immunostaining was then performed. Invasive areas with strong desmoplastic fibrosis, identified by expert pathologists (N.Y. and T.S.) were used for evaluation of immunohistochemical expression. Twelve tissue cores (3 mm thick; 10 cancer tissues, 2 control tissues) were obtained from target lesions and were used to prepare recipient blocks. In addition, desmoplastic tissues surrounding invasive colorectal cancer and interstitial lung disease were evaluated as positive controls. Subsequently, 3-μm-thick sections were prepared, and hematoxylin and eosin staining was performed to confirm the histological diagnosis. Serial sections obtained from TMA blocks were employed for immunohistochemical analysis.

***Immunohistochemistry***

Three-micron-thick tissue sections were mounted on charged slides, and the slides were dried and melted at 62°C for 20 min. Sections were deparaffinized, rehydrated, heated for 20 min in Envision FLEX target retrieval solution (pH 6.0 or 9.0; Dako), and washed twice (5 min each) in phosphate-buffered saline (PBS). Next, endogenous peroxidase activity was blocked using hydrogen peroxide (3%) for 5 min, and nonspecific binding was blocked by treatment with 1.5% normal serum in PBS for 35 min at room temperature. **Supplementary Table 1** lists the antibodies used in this study. The DAKO Envision+ system was used for immunohistochemical analysis. Briefly, specimens in citrate buffer (pH 6.0) were heated using a microwave (H2500; Microwave Processor; Azumaya) 3 times at 750 W (5 min each time) and then incubated with antibodies. The antigen-antibody reaction was visualized using an enhanced polymer-based detection system, and counterstaining was performed using hematoxylin.

**Reference**

1. Hayakawa K, Ed. (2014). Guidelines for Diagnosis and Treatment of the Lung Cancer. Kanehara Co. Ltd.
